# The last decade epidemiologic concern of drinking water contaminants of emerging concern (CECs) in Asian Countries: A scoping review

**DOI:** 10.1016/j.heliyon.2024.e39236

**Published:** 2024-10-12

**Authors:** Rinaldy Jose Nathanael, Latonia Nur Adyanis, Katharina Oginawati

**Affiliations:** a Environmental Engineering Program, Faculty of Science and Technology, Airlangga University, Surabaya, 60115, Indonesia; b Department of Environmental Engineering, Chung Yuan Christian University, Taoyuan, Taiwan, 320314; c Environmental Management Technology Research Group, Department of Environmental Engineering, Faculty of Civil and Environmental Engineering, Bandung Institute of Technology, Bandung, 40132, Indonesia

**Keywords:** Scoping review, Emerging contaminants, Drinking water, Epidemiology, Asia

## Abstract

With the rapid industrialization and urbanization in Asian countries, the challenge of rising emerging contaminants in the environment, including the water cycle, has become more pronounced. Consequently, the presence of CECs in drinking water systems is inevitable due to their ubiquitous nature in aquatic environments. This scoping review aims to identify epidemiological concerns regarding drinking water CECs in Asian countries over the past decade by describing the types of assessed CECs, their associated health effects, and identifying gaps and future research prospects through a summary of relevant studies. Searches were conducted on PubMed and Scopus up to February 29, 2024. Included were epidemiological studies from the past 10 years (since January 2014) in Asian countries that assessed emerging contaminants in drinking water through direct measurement or analysis as factors. From an initial pool of 3198 results, 15 relevant studies were selected. These studies assessed various types of CECs, including disinfection byproducts (n = 10), endocrine disruptors (n = 2), pesticides (n = 2), and a protozoan pathogen (n = 1). The meticulous assessment of CECs and associated health outcomes in Asian epidemiological studies over the past decade has been deemed inadequate to address the wide range of ubiquitous CECs in drinking water and their potential health effects that have not yet been addressed. While not the sole objective, the primary aim of epidemiological studies is to inform policy decisions and increase awareness among the public and policymakers. Therefore, researchers in Asian countries, particularly in environmental and public health fields, should prioritize the development of research in this area by exploring more CECs type and associated health outcomes.

## 1 Introduction

Water quality and accessibility are universal concerns that are integral to global well-being and human rights. Access to clean and safe drinking water is not merely a matter of convenience, but a pivotal determinant of public health [1]. This responsibility transcends borders and resonates with the aspirations of Sustainable Development Goal 6 (SDG 6), which emphasizes the global mission for water sustainability. The persistent lack of quality or accessibility of drinking water remains a primary concern, contributing to the failure of public health, not to mention its individual health impacts.

Untreated or inadequately treated drinking water may cause gastrointestinal problems [[2], [3], [4]], bacterial [[3], [4], [5]] and virus [[6], [7], [8]] infections, high blood pressure [[9], [10], [11]], cardiovascular problems [[12], [13], [14]], carcinogenic effect [[15], [16], [17]], and other various diseases [[18], [19], [20], [21]]. Inferring the causal relationship between a disease and its cause, including waterborne diseases and drinking water, is one of the purposes of epidemiological studies, which seeks to comprehend the distribution and determinants of health-related events, allowing for a comprehensive understanding of the factors influencing the well-being of communities [22,23].

The global landscape of drinking water epidemiology has evolved significantly since the initial explorations into waterborne diseases. Early concerns primarily centered around chemicals [[24], [25], [26]] and waterborne pathogens [27,28] giving rise to pivotal advancements in water treatment and sanitation. Today, it encompasses a broader range of health outcomes, including those associated with chronic conditions [10,11,16] and emerging contaminants [[29], [30], [31]]. Emerging Contaminants or Contaminants of Emerging Concern (CECs) are pollutants detected in environmental monitoring samples that may cause ecological or human health impacts, which are not commonly monitored or not regulated under current environmental laws [32,33]. Sources of these pollutants include agriculture, urban runoff, and ordinary household products such as soaps, disinfectants, and pharmaceuticals [33,34]. CECs can enter the water cycle through various processes such as runoff into rivers, direct effluent discharge, or seepage and infiltration into the water table [34,35]. Some CECs are known to cause endocrine disrupting activity [36,37] and other toxic mechanisms [38,39]. They can be broadly classified into several categories of chemicals such as pharmaceuticals, personal care products, cyanotoxins, nanoparticles, and flame retardants [40].

Epidemiological studies play a crucial role in addressing the challenge of determining the burden of diseases associated with emerging contaminants (CECs) in drinking water. Several studies have identified the presence of emerging contaminants in drinking water and their potential health impacts across Asia, such as disinfection byproducts on semen quality [41] and kidney function [42], endocrine disrupting chemicals on hepatic function [43], and organochlorine pesticides on diabetes [44]. However, there remains a notable lack of comprehensive reviews that integrate such findings to identify gaps, challenges, and future research directions. This gap in research is significant given the unique environmental, social, and regulatory contexts in Asia that could influence the presence and impact of CECs differently than in other regions. Therefore, the primary aim of this review is to bridge this gap and address emerging contaminants in drinking water in Asian countries as a whole. This will be achieved by summarizing relevant research from the last decade, integrating these findings to highlight key results and gaps, and providing a comprehensive overview of the epidemiological landscape related to CECs in drinking water.

This scoping review aims to summarize the epidemiology research of CECs in drinking water in the last 10 years in Asian Countries to capture the current state of knowledge and concerns in CECs in drinking water and to identify knowledge gaps and prospects for future research. Specifically, the review will highlight: (1) an overview of the types of assessed CECs associated with drinking water, (2) The potential health risks and diseases that may be associated with CECs in drinking water, (3) the challenges of managing emerging contaminants in drinking water in Asia, and (4) Recommendations from the studies and future challenges. By consolidating findings from various sources, the review will contribute to the establishment of a more unified and coherent body of knowledge on the epidemiology of CECs in drinking water. This, in turn, may inform public health policies, regulatory frameworks, and future research directions aimed at preventing the adverse health effects associated with these contaminants.

## 2 Method

The review was structured following the Preferred Reporting Items for Systematic Reviews and Meta-Analyses–Extension for Scoping Reviews (PRISMA-ScR) statement guidelines, aiming to conduct a thorough and impartial exploration of relevant literature [45].

To collect relevant data about epidemiology research on CECs in drinking water, we conducted a thorough literature search to retrieve relevant articles from PubMed and Scopus databases. We limit the search to PubMed and Scopus to ensure that the included studies are of high quality, peer-reviewed, and methodologically sound, aligning with the specific needs of an epidemiological scoping review. The retrieved articles were screened using online systematic review software Covidence. The titles and abstracts of every publication underwent screening and were cross-referenced against the inclusion criteria for a detailed examination of the full text. Only peer-reviewed literature written in English and met all other inclusion criteria in Table 1 was considered for inclusion.

**Table 1:** Inclusion criteria.

Criteria	Evidence
Language	Written in English
Exposure Context	Drinking water context: epidemiology of disinfection byproducts OR other CECs evidenced by measurement of CECs concentration in water OR drinking water source as one of the assessed factors in surveys
Cause of Disease/Outcome	Discuss at least one pollutant considered as CECs
Study Design	Epidemiological study design
Location	Asian Countries setting

The complete search logic (search within title, abstract, and keywords) are: (“Drinking Water” OR “Tap Water” OR “Water Quality” OR “Water Contamination”) AND (“Disease” OR “Health Impact” OR “Health Risk” OR “Health Outcome” OR “Epidemi∗" OR “Public Health” OR “Population Health” OR “Case∗" OR “Cohort” OR “Cross section∗") AND (“Emerging” OR “CEC∗” OR “PFAS” OR “PCB” OR “PBB” OR “PBDE” OR “DBP∗” OR “Retardant” OR “Endocrine Disruptor” OR “Microplastic∗" OR “Nanoplastic∗” OR “Pharmaceutic∗" OR “PPCP∗” OR “Cyanotoxi∗” OR “Pesticide∗”). The asterisk (∗) sign serves as a wildcard character. It represents any group of characters, including no character. The types of literature retrieved are articles and conference proceedings only. Inclusion criteria for the studies encompassed three main categories of environmental epidemiology [46] to ensure a comprehensive examination of the literature. These categories are: descriptive epidemiology, inferential epidemiology, and ecological/correlational studies.

Two reviewers independently extracted/charted data from each eligible article and merge them afterwards using online systematic review software Covidence. Any disagreements were resolved through discussion between two reviewers and further adjudication by a third reviewer. Data on basic article characteristics such as contaminants, countries, and health effects were abstracted and summarized to tables and charts. The drinking water context & challenges were also scrutinized for evidence synthesis.

## 3 Results

### 3.1 Study selection

**Fig. 1:**
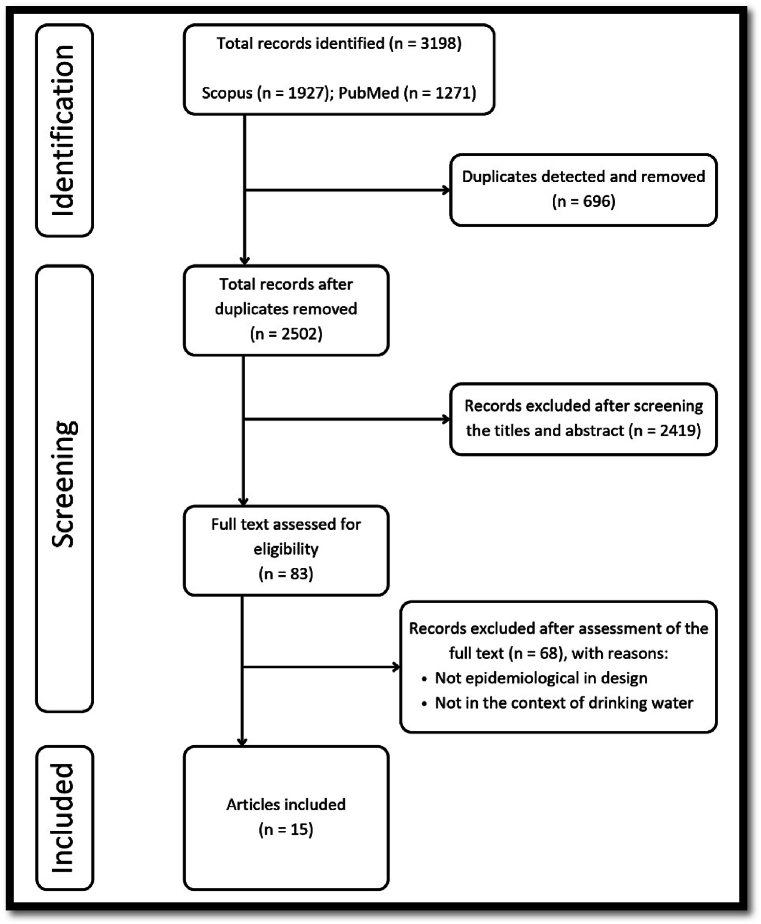
PRISMA flowchart of the article selection process.

A total of 3198 records were initially retrieved from two databases, Scopus and PubMed. 696 duplicate records were identified and eliminated. Subsequently, 2502 studies underwent screening based on their titles and abstracts. From this screening process, 83 studies were deemed eligible for full-text assessment to determine their suitability for inclusion in this review. After a thorough assessment of the full texts, 68 studies were excluded due to their non-epidemiological design or lack of relevance to the context of drinking water. Ultimately, a total of 15 studies were included in this review (See Fig. 1 for PRISMA flowchart). The included studies are summarized in more detail in Table 2. The agreement on study inclusion outcomes was reached by both independent reviewers and there were no remaining conflicting perspectives between the two reviewers. Out of the 15 records encompassed in this study, 12 (80 %) originated from China. Additionally, individual studies were conducted in Sri Lanka, the Philippines, and India, totaling three studies in each of these respective countries. All of the records included were in the form of peer-reviewed journal articles.

**Table 2:** Summary of the basic characteristics of included studies published from Jan 2014 to Feb 2024, sorted by date.

No	Author	Year	Country	CEC Assessed	Outcome	Study Design
1	Zeng et al. [41]	2014	China	Disinfection Byproducts (DBPs)	Semen Quality	Cross-sectional study
2	Jayasumana et al. [47]	2015	Sri Lanka	Herbicide	Chronic Kidney Disease of Unknown Etiology (CKDu)	Case control study
3	Cao et al. [48]	2016	China	Disinfection Byproducts (DBPs)	Fetal Growth & Gestational Age	Cohort study
4	Labana et al. [49]	2018	Philippines	Cryptosporidium Oocysts	Cryptosporidium infection	Case control study
5	Yin et al. [50]	2019	China	Polybrominated diphenyl ethers (PBDEs)	Birth outcome	Cohort study
6	Zhang et al. [51]	2019	China	Disinfection Byproducts (DBPs)	High blood pressure	Cross-sectional study
7	Zhang et al. [52]	2021	China	Disinfection Byproducts (DBPs)	Blood Pressure and Platelet indices	Cross-sectional study
8	Tyagi et al. [44]	2021	India	Organochlorine Pesticides	Type 2 Diabetes	Case-control study
9	Fu et al. [43]	2022	China	Endocrine Disrupting Chemicals	Hepatic function	Cross-sectional study
10	Deng et al. [53]	2022	China	Disinfection Byproducts (DBPs)	Ovarian reserve	Cross-sectional study
11	Deng et al. [54]	2022	China	Disinfection Byproducts (DBPs)	Menstrual cycle characteristics	Cross-sectional study
12	Deng et al. [55]	2023	China	Disinfection Byproducts (DBPs)	In Vitro Fertilization Outcomes	Cohort study
13	Luo et al. [56]	2023	China	Disinfection Byproducts (DBPs)	Birth outcome	Cohort study
14	Liu et al. [57]	2024	China	Disinfection Byproducts (DBPs)	Diminished ovarian reserve	Case-control study
15	Li et al. [42]	2024	China	Disinfection Byproducts (DBPs)	Kidney Function	Cross-sectional study

### 3.2 Included studies basic characteristics

**Fig. 2:**
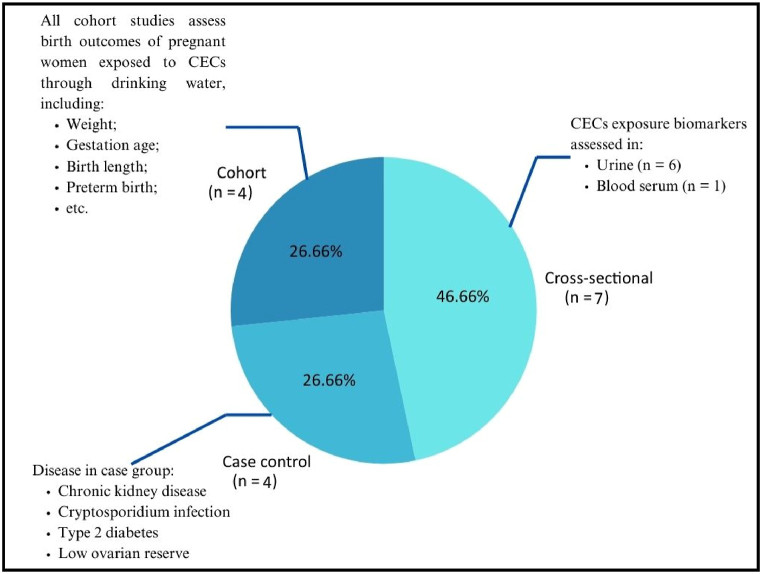
Study type of the included studies.

**Fig. 3:**
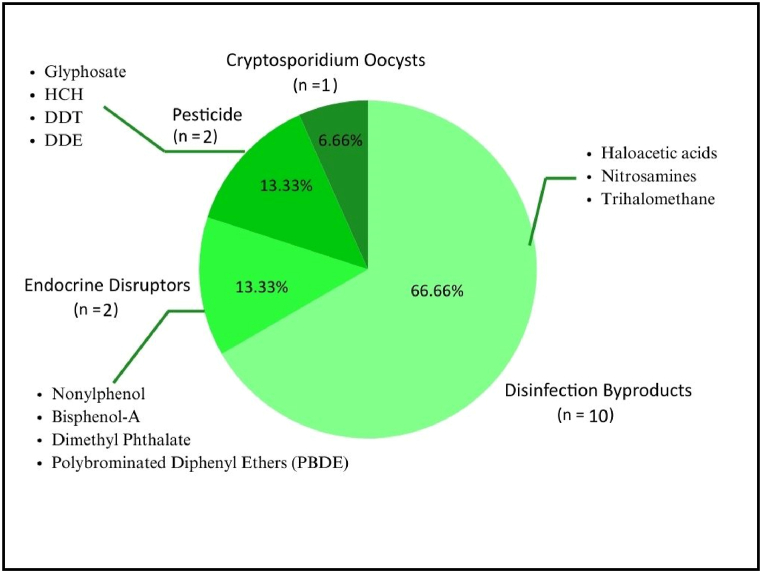
CECs type of the included studies.

**Fig. 4:**
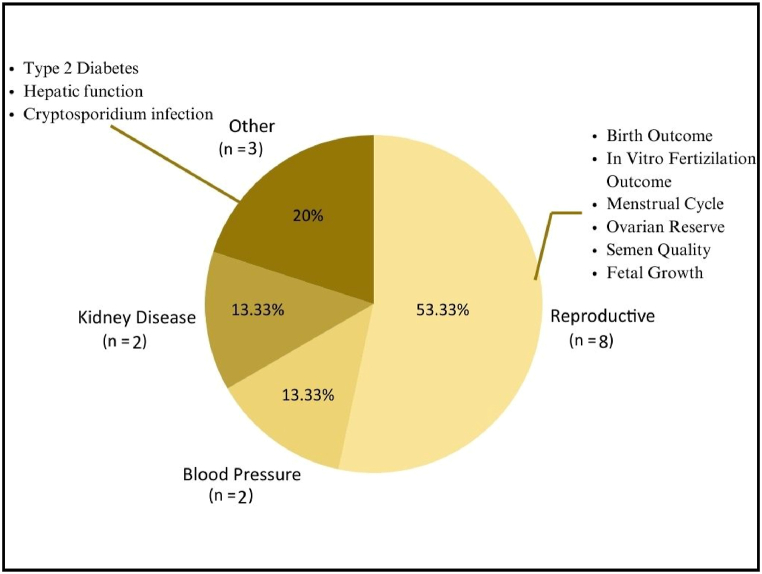
Health outcomes of the included studies.

The included epidemiology studies have different design, type of CECs assessed, and type of health outcomes assessed. These basic characteristics are depicted in pie charts in Fig. 2, Fig. 3, and Fig. 4. In the analysis of 15 epidemiological studies, 7 (46.66 %) employed a cross-sectional design, 4 (26.66 %) were case-control studies, and 4 (26.66 %) were cohort studies. In the case-control studies, participants were divided into two groups: the control group and the case group, representing individuals with chronic kidney disease of unknown etiology, those positive for cryptosporidium infection, individuals positive for type 2 diabetes, and those with low ovarian reserve, respectively. Biomarkers from urine and blood serum were collected from participants in the included cross-sectional studies to assess their exposure to emerging contaminants of concern. In all of the cohort studies included, pregnant women visiting the hospital were recruited and followed up until delivery, with data on birth outcomes recorded.

Out of 15 studies, 10 (66.66 %) assess emerging contaminants classified as disinfection byproducts (DBPs). The DBPs assessed were varied, mainly haloacetic acids (HAAs) such as dichloroacetic acid (DCAA) and trichloroacetic acid (TCAA), which constitute 8 (80 %) of the 10 studies about DBPs. Additionally, two studies (13.33 %) focused on investigating endocrine disrupting chemicals (EDCs), including nonylphenol, bisphenol A (BPA), dimethyl phthalate, and polybrominated diphenyl ethers (PBDE), with each study exploring different combinations of these substances. Furthermore, two studies (13.33 %) examined the presence and effects of pesticides, specifically glyphosate, hexachlorocyclohexane (HCH), dichlorodiphenyltrichloroethane (DDT), and its metabolite dichlorodiphenyldichloroethylene (DDE). While classified separately here, these pesticides can also be considered EDCs and they will also be discussed both jointly and separately from EDCs. Lastly, one study investigated the presence of cryptosporidium oocysts in drinking water sources and its association with cryptosporidium infection cases.

Based on the disease/outcome assessed in each study, 8 (53.33 %) of the included records explored the relation between drinking water contaminants of emerging concern (CECs) and reproductive health outcomes. These encompassed assessments of semen quality, menstrual cycle patterns, ovarian reserve, birth and in vitro fertilization (IVF) outcomes, and fetal growth. Notably, a significant proportion of the study populations comprised patients from fertility clinics and pregnant individuals. Furthermore, 2 (13.33 %) records assessed the association of drinking water CECs and blood pressure, while another 2 delved into their association with kidney disease. Three additional records explored various other effects attributable to these contaminants.

All of the included studies measured the concentration of the emerging contaminants assessed in each study. However, these concentrations were measured in various compartments including drinking water, blood, blood serum, urine and colostrum. The reported average concentrations of CECs across all studies are summarized in Appendix Table 1.

## 4 Discussion

### 4.1 The assessed emerging contaminants

#### 4.1.1 Disinfection byproducts (DBPs)

DBPs are a significant category of contaminants that warrant attention. DBPs are organic and inorganic compounds that result from chemical reactions between organic and inorganic substances such as contaminants and chemical treatment disinfection agents, respectively, during water disinfection processes [58]. By that definition, DBPs are the type of emerging contaminants exclusively and commonly found in treated drinking water. This might be the reason that this type of CEC dominates the included studies (66.67 %). DBPs arise from the interaction between disinfectants such as chlorine and naturally occurring organic substances in water. This reaction occurs mainly in disinfected drinking water and swimming pools treated with chlorine and chloramine. Natural organic matter, including fulvic and humic acids, amino acids, as well as iodide and bromide ions, can react with disinfectants to yield a diverse array of DBPs [59]. More than 600 DBPs have been identified [60], but only several classes are generally well-known and discussed, such as trihalomethanes (THMs) and haloacetic acids (HAAs), which are the DBP classes formed at the highest concentrations after chlorination [58]. They constitute approximately 25 % of the identified halogenated DBPs [60].

Out of 10 included study results about DBPs, 8 specifically investigated the HAAs class, such as TCAA and DCAA [41,42,[51], [52], [53], [54], [55],^57^], one investigated THMs [48], while another one investigated nitrosamines [56]. Given previous findings suggesting that TCAA and DCAA serve as more reliable biomarkers for assessing disinfection byproduct (DBP) ingestion, particularly from chlorinated drinking water sources [41], it is unsurprising that 80 % of the studies included in our analysis specifically investigated the HAAs) class, with TCAA and DCAA as primary focus. This preference for TCAA and DCAA as biomarkers over THMs can be attributed to the rapid metabolism of THMs in the body following ingestion, inhalation, and dermal contact, which can compromise the accuracy of their measurement. Conversely, TCAA and DCAA exhibit a longer half-life in the body and demonstrate a significant correlation between their ingestion via drinking water and urinary excretion, as highlighted in the study [41]. As understanding exposure pathways is crucial, each study specified that the research population potentially faced exposure through the consumption of tap water or water from distribution systems, as well as through activities involving chlorinated water, such as bathing, showering, or swimming. This is particularly important considering that blood concentrations were noted to be strongly influenced by very recent exposure, with showering and bathing demonstrating a more pronounced effect on blood levels compared to other water-use activities. Previous studies [61,62] demonstrated that blood THM concentrations decrease steadily within 30 min post-shower or bath, offering a window into steady-state levels. These findings emphasize the significance of short-term exposure dynamics in understanding blood THM levels, especially concerning routine water-related activities like bathing and showering. These studies have provided valuable insights into the potential risks associated with exposure to these prevalent DBPs, contributing significantly to the understanding of their health effects and informing regulatory standards and water treatment practices. However, it is essential to recognize that different chemical species of DBPs may exhibit unique chemical properties and toxicity [63,64] which could result in varying health effects.

In the included results of this study, DBPs are associated with reproductive function, including ovarian reserve [53,57] menstrual cycle [54], semen quality [41], birth outcomes [48,56], and IVF outcomes [55]. Only 2 studies linked DBPs to blood pressure [51,52], and only one study examined their impact on kidney function [42]. The majority (70 %) of epidemiological studies of DBPs included in our review focused primarily on reproductive function. The authors opine that the health outcomes of DBPs assessed in the included studies lack breadth, when compared to previous epidemiological studies conducted outside of Asian countries and other non-epidemiological studies addressing this issue. Epidemiological evidence consistently links long-term exposure to THMs with an increased risk of bladder cancer, although causality remains inconclusive and evidence regarding other cancer sites is mixed [58]. Populations highly exposed to swimming pools exhibit respiratory symptoms and asthma, but the direction of association is unclear [58]. Another study focuses on DBPs in chlorinated drinking water, categorizing them into aliphatic, alicyclic, and aromatic types, which exhibit cytotoxicity, mutagenicity, and carcinogenicity [65]. Long-term exposure is associated with carcinogenic, reproductive, and developmental effects. All of these potential effects have yet to be explored in Asian countries through epidemiological studies. For example, a previous meta-analysis of case-control studies on disinfection byproducts and bladder cancer in Europe and North America reported a significant odds ratio (OR = 1.47, 95 % CI = 1.05–2.05) for men exposed to average residential total trihalomethanes (TTHM) levels greater than 50 μg/l [66]. Findings like this are highly valuable, not only locally but globally. However, it is important to note that water disinfection practices, and consequently the occurrence of DBPs, differ across countries. This variation underscores the relevance and need for local epidemiological studies to better understand and address region-specific risks associated with DBP exposure.

While the assessed reproductive health endpoints being assessed in the included studies are undoubtedly important and warrant investigation, the predominance of research in this area may overlook other potentially significant health effects associated with DBP exposure. This disparity between the health outcomes explored in epidemiological research and the broader spectrum of health effects documented in toxicological studies highlights the need for greater diversification and expansion of research efforts in this field. Future epidemiological studies should aim to investigate a wider range of health outcomes, including those identified in toxicological and animal studies [58]. Moreover, the potential for interaction effects and additive toxic effects between different DBP chemical species cannot be overlooked. Therefore, while existing research has primarily focused on addressing the health impacts of common DBP classes, future investigations should broaden their scope to explore other chemical species of DBPs comprehensively.

#### 4.1.2 Endocrine disrupting chemicals (EDCs)

The term ‘endocrine disrupting chemicals’ (EDCs) covers a wide array of exogenous substances that interfere with the endocrine system's normal function, often by mimicking or blocking hormones, thereby disrupting hormonal balance and physiological processes [67]. These compounds encompass a diverse range of chemicals, including but not limited to bisphenol A (BPA), phthalates, dioxins, and polychlorinated biphenyls (PCBs), all cited for their ability to mimic endogenous hormones, block hormonal signals, or alter the natural production and breakdown of hormones [68,69]. The sources of these disruptors are equally varied, spanning from industrial discharges, pesticides, and plastics to personal care products, underscoring the pervasive nature of these chemicals in the environment [67]. When endocrine disruptors are present in water bodies used as raw water for drinking water, the existing conventional water treatment plant may not effectively remove them [70,71].

There are a total of 4 results included in this study that discuss substances that may be categorized as endocrine disruptors. Various chemicals were assessed, including nonylphenol, BPA, dimethyl phthalate [43], flame retardants such as polybrominated diphenyl ethers (PBDE) [50], and pesticides (which will be discussed in more detail in the next section, for its unique issue and source) such as glyphosates [47], hexachlorocyclohexane (HCH), dichlorodiphenyltrichloroethane (DDT), and its breakdown product dichlorodiphenyldichloroethylene (DDE) [44]. While the assessed EDCs provide a significant step in addressing this type of emerging contaminant in drinking water in Asia, it's crucial to acknowledge that the realm of EDCs is vast and continually evolving alongside industrial and agricultural advancements. The diversity and the wide range of sources from which EDCs emanate signify that the chemical substances reviewed here may only represent a fraction of the potential EDCs impacting drinking water and public health. One particular study by Yin [50] found that colostrum samples from areas with highly intensive construction material production exhibited higher concentrations of PBDEs. This correlation was particularly notable when a higher percentage of women in these areas reported using tap water as their primary drinking water source.

In the included results of this study, EDCs are associated with birth outcomes [50], diabetes [44], hepatic function [43], and chronic kidney disease [47]. Extensive documentation has linked exposure to EDCs with a range of adverse health effects, encompassing developmental, reproductive, neural, immune, and metabolic disturbances [72]. There's growing evidence that endocrine disruptors contribute to the increasing incidence of diseases such as obesity, diabetes, and various cancers, by interfering with hormone function [73]. Furthermore, the vulnerability of certain populations, like children and pregnant women, elevates the concern surrounding these disruptors due to the potential for long-lasting effects from exposure during critical windows of development [73]. Although lacking depth for only one study found for each associated effects, the breadth of health outcomes explored in the included studies concerning EDCs demonstrates a notable advancement compared to the epidemiological research on DBPs in drinking water across Asia over the past decade. The inclusion of various health endpoints associated with EDCs exposure, such as birth outcomes, diabetes, hepatic function, and chronic kidney disease, indicates a more comprehensive understanding of the potential health impacts of EDCs. Still, other health effects of EDCs yet to be explored, such as obesity, immune response, or cancer, are a broad research field prospect to be considered in future research to bring the EDCs into the awareness of policymakers and the public in Asian countries alike.

Future research should try to explore additional endocrine-disrupting chemical species, particularly those with increasing environmental and biological prevalence but lesser-known health impacts. For instance, perfluoroalkyl and polyfluoroalkyl substances (PFAS), often referred to as “forever chemicals” due to their persistent nature, are emerging as significant concerns for endocrine disruption [74]. Additionally, newer forms of bisphenols such as bisphenol S (BPS) and bisphenol F (BPF), which are being used as alternatives to BPA, warrant closer scrutiny for their endocrine-disrupting potentials [75]. Furthermore, the expansion of nanotechnology introduces nanomaterials as potential EDCs, raising questions about their environmental health safety given their unique properties and interactions with biological systems [76].

Future research could also benefit by exploring rural and semi-urban parts of Asian countries, where endocrine-disrupting chemicals (EDCs) might be present in drinking water. Contrary to the common notion that EDCs in the environment are primarily linked to industrial and urban activities, a previous study on bisphenol A (BPA) suggests that these contaminants can also be detected in areas with less industrial development [77]. This finding underscores the importance of investigating diverse geographic settings to fully understand the prevalence and impact of EDCs in drinking water across different communities. Expanding research to include these areas would provide a more comprehensive understanding of EDC exposure and its potential health effects in Asian populations.

#### 4.1.3 Pesticides

Pesticides, substances designed to prevent, destroy, or control pests, encompass a broad range of chemical classes, including organophosphates, carbamates, pyrethroids, and neonicotinoids, each with distinct mechanisms of action and applications [78]. The use of pesticides in Asian agricultural practices continues to pose significant concerns in terms of environmental pollution and human health hazards, despite efforts to phase out the most toxic and hazardous pesticides. The persistence of other toxic classes of pesticides in the region underscores a critical gap in pesticide management and regulation. Sources of human exposure to pesticides are equally diverse, spanning occupational settings, such as farming, to residential exposure through food and water consumption and proximity to treated areas. Pesticides can find their way into drinking water through various pathways, such as surface runoff, leaching, and groundwater drainage [79]. Studies conducted across various Asian countries have highlighted the ubiquitous presence of pesticides and their breakdown products in water bodies and treatment plants [80,81]. For instance, a previous review study [82] confirmed the widespread contamination of water sources with pesticides in South Asian countries, linking this phenomenon to agricultural runoff and inadequate waste management practices. This widespread contamination signals potential risks to drinking water quality and, subsequently, public health.

Only two significant studies conducted in Sri Lanka and India [44,47] over the last decade focusing on this critical area. These two studies have successfully associated the exposure to chronic kidney disease [47] and diabetes [44], confirmed the threat of pesticides in drinking water. Study in India [44] shows that the samples from ground water and tap water samples have slightly higher organochlorine pesticides (OCPs) concentration than the permissible limit. Furthermore, drinking water consumed by the diabetes group has the highest OCPs level. Meanwhile, in Sri Lanka [47], over 94 % abandoned wells in study areas were contaminated with glyphosate, while those male farmers who have a history of drinking from these abandoned wells were at significantly higher risk of developing CKDu. The two studies highlighted a glimpse into the potential health impacts of drinking water contaminated with pesticides. Yet, these instances barely scratch the surface of the possible spectrum of health outcomes of pesticides still used presently in Asian countries [83]. The scarcity of epidemiological research on the impact of pesticide contamination in drinking water in Asia further compounds the issue.

Additionally, a previous meta-analysis assessing health risks due to exposure to pesticide residues in drinking water highlighted that some Asian countries, particularly the Philippines and China, had a hazard index exceeding 1, indicating a non-carcinogenic threat to human health [84]. As a meta-analysis, the assessment was limited to the available studies in each region. This study exemplifies how health risks due to pesticide exposure from drinking water are a crucial yet underexplored topic in Asian countries.

The health effects associated with pesticide exposure have been a focus of extensive research, particularly focusing on other exposure pathways, such as inhalation, as well as through toxicological studies, beyond the context of drinking water. Those research revealed both acute and chronic outcomes that need further confirmation in the context of exposure from drinking water. Short-term, high-level exposure, often occurring in occupational settings, has been linked to acute poisoning symptoms, ranging from nausea and dizziness to severe neurotoxic effects and death [85]. Chronic exposure, even at low levels, is associated with a range of long-term health issues, including neurodegenerative diseases, such as Parkinson's disease, various forms of cancer, reproductive and developmental toxicity, and endocrine disruption [86,87]. The health risks posed by pesticides underscore the importance of regulatory measures, appropriate use practices, and ongoing research to minimize water pollution, mitigate exposure, and develop water treatment technology.

Heightened public awareness and regulatory scrutiny contrast with limited recent epidemiological evidence, posing a challenge in addressing pesticide contamination in Asian drinking water. Despite pesticides being more regulated and widely recognized first [88] compared to other contaminants like disinfection byproducts (DBPs) and endocrine-disrupting chemicals (EDCs), the research landscape reveals a significant disparity in the breadth and depth of studies conducted. This scenario suggests an urgent need for a concerted research agenda that bridges this knowledge gap. It's crucial to broaden epidemiological studies to cover a wider range of pesticides and their breakdown products due to evolving agricultural practices and the introduction of new chemicals that may pose unknown health risks [89].

#### 4.1.4 Cryptosporidium oocysts

Cryptosporidium, a protozoan parasite, is increasingly recognized as a significant public health concern due to its presence in food, water, and the environment, and the robust nature of its oocysts, which are resistant to conventional water treatment methods, such as chlorination [90]. These oocysts, which are released in the feces of infected hosts, can survive in diverse environmental conditions. Their small size and resilience enable them to contaminate drinking water sources, including rivers, lakes, and groundwater [91]. Contamination occurs through various routes, such as human and animal fecal matter entering water sources via sewage overflow, wastewater treatment plants discharge, and agricultural runoff [91]. Once ingested, even in small quantities, Cryptosporidium oocysts can cause cryptosporidiosis in humans, an illness characterized by severe diarrhea, dehydration, and weight loss, with potentially fatal outcomes in immunocompromised individuals [92].

Recent studies have highlighted an increasing prevalence of Cryptosporidium in Asia, attributing the rise to factors such as water contamination and inadequate sanitation [93]. In particular, a study by Feng et al. [94] underscored the genetic diversity of Cryptosporidium species in China, pointing to the complexity of controlling its transmission among humans and animals. This diversity encompasses multiple species and genotypes, each with different host specificities, environmental resilience, and resistance to treatment methods, thereby complicating efforts to implement uniform control measures. The detection of Cryptosporidium in drinking water sources in India further emphasizes the challenges in ensuring water quality and the potential risk of waterborne outbreaks in populous regions [95]. Furthermore, certain cultural and agricultural practices unique to Asia exacerbate the contamination of water bodies. For instance, the common practice of using untreated or inadequately treated animal manure as fertilizer for crops can lead to runoff contaminating water sources. Such runoff is particularly concerning during the monsoon season, prevalent in many Asian countries, which can spread contaminants over a wide area, affecting multiple water sources [96]. Additionally, the proximity in which people and livestock coexist in rural and semi-urban areas of Asia facilitates the zoonotic transmission of protozoan parasites, complicating the efforts to control their spread [97].

Despite the growing detection and increasing awareness of Cryptosporidium's presence in Asia, epidemiological studies focusing on this pathogen in drinking water are surprisingly sparse. The scarcity of detailed epidemiological research is exemplified by the limited investigation, with only a notable study by Labana et al. [49] delving into the epidemiological landscape of Cryptosporidium infections in the Philippines. In this study, drinking water sources were found to be a significant risk factor, alongside open pit toilets, affecting the risk of infection. This study highlighted the significant yet underreported burden of cryptosporidiosis, suggesting a similar trend might be present in other Asian countries [98].

While only one epidemiological study concerning cryptosporidium in Asian drinking water has been identified in the past decade, from the author's perspective, its status as an emerging contaminant and pathogen is less alarming than the three chemical contaminants discussed earlier. There are several reasons for this. Firstly, its naturally occurring nature sets it apart from anthropogenic chemicals, making it widely recognized through numerous case reports (though not epidemiologically designed) [98]. Secondly, its specific health effects facilitate easier diagnosis as a causal agent. Furthermore, these effects – infection symptoms – are reversible with treatment, unlike the toxic effects of chemical contaminants, that are harder to diagnose because they might cause a variety of clinical signs and symptoms [99] and might have irreversible damage. Thirdly, water treatment methods for cryptosporidium are as simple yet effective as boiling, readily applicable, and already practiced by many consumers in Asian countries [100]. This contrasts with chemical contaminants, which may not be effectively removed through boiling alone. Furthermore, infrastructure and health systems may be the most influential factors contributing to the prevalence of this disease. Therefore, enhancing these factors could lead to improvements in disease management and prevention.

However, it is important to note that Cryptosporidium is a protozoan pathogen, unlike common bacteria routinely monitored at water treatment plants and regulated accordingly (hence its status as an emerging pathogen due to its unregulated and current threat). Previous studies in Europe have recognized the often-neglected threat of Cryptosporidium in drinking water, noting its detection in the drinking water supply system [101] and its role in causing an outbreak in another European country [102]. Both detection and epidemiological studies like these are valuable for local actions, which are still lacking in Asian countries. Therefore, Cryptosporidium and other waterborne protozoan pathogens, like Giardia, Toxoplasma, Entamoeba, Cyclospora, Isospora, Blastocystis, Balantidium, Acanthamoeba, Sarcocystis, and Naegleria [91] are worth mentioning here as significant emerging contaminants/pathogens that must be addressed in future research endeavors to enhance public health awareness regarding emerging contaminants in drinking water.

### 4.2 Health outcomes

A more detailed explanation of the emerging contaminant substances included in the studies and their subsequent health effects indicators are presented here (Table 3). From the results and summary provided, it is evident that some of the included studies, considered together, successfully captured the causal relationship between contaminants exposure and the subsequent health effects, especially for the DBPs and reproductive function [41,48,[53], [54], [55], [56], [57]], since the results of the association between the exposure and effect biomarkers are constantly significant. On other CECs however, there were not enough similar studies to confidently infer causality, despite a significant association between the exposure and health effects being found. It is apparent in this case that only one epidemiology study about a specific cause and effect is inadequate to confidently infer causality. Determining if a noted statistical link signifies a causal relationship between exposure and subsequent health effects necessitates conclusions that extend well beyond the data from one study. It also involves taking into account other factors, such as the strength of the association, the agreement of results from various studies, and the biological plausibility [103]In addition to the main findings, a supplementary table (Appendix Table 1) summarizes the concentrations of contaminants of emerging concern (CECs) found in water and biological compartments, such as urine, blood, and colostrum, across the included studies. In the study conducted in Asia, the maximum urinary concentration of trichloroacetic acid (TCAA) was 9.58 μg/L, indicating a relatively high level of exposure. In comparison, data from Spain revealed a significantly lower concentration of 0.12 μg/L [104], while the UK study reported an intermediate level of 6.06 μg/L [105]. These variations suggest that environmental and regulatory differences across regions may contribute to disparate TCAA exposure levels, with the Asian cohort exhibiting the highest measured concentration.

**Table 3:** Non-exhaustive summary of drinking water CECs and health effects in the included studies, sorted by date.

No	Author	Year	Country	CEC Assessed^a^	Outcome^a^	Brief Summary
1	Zeng et al. [41]	2014	China	Trichloroacetic acid (TCAA)^b^	Sperm count, concentration, motility, and %normal morphology	Increased adjusted odds ratios (ORs) are observed for men with elevated urine TCAA concentration compared to men with urine TCAA in the lowest quartile. Exposure to drinking-water DBPs may contribute to decreased semen quality in humans.
2	Jayasumana et al. [47]	2015	Sri Lanka	Glyphosate^c^^**,**^^d^	Case of Chronic Kidney Disease of Unknown Etiology (CKDu)	This case-control study strongly supports the hypothesis that CKDu in Sri Lanka is a drinking-water-related disease in farmers who have a history of spraying glyphosate.
3	Cao et al. [48]	2016	China	Trihalomethanes^b^	Birth weight & length, gestational age, and small for gestational age (SGA) case	Elevated maternal blood THM concentrations were associated with decreased birth weight, reduced birth length, and increased risk of SGA, suggesting that elevated maternal THM exposure during late pregnancy may adversely affect fetal growth.
4	Labana et al. [49]	2018	Philippines	Cryptosporidium Oocysts	Cryptosporidium infection	Seven (29.2 %) of the 24 water samples were positive for oocysts with highest conc. of 0.8 oocyst/L. 3 significant risk factors were found: location, drinking water, and use of open pit sanitary facility.
5	Yin et al. [50]	2019	China	Polybrominated diphenyl ethers (PBDEs)^c^	Birth weight, head circumference	Maternal age and drinking water source were identified as the major influencing factors for the PBDE levels in colostrum samples. The tri-to penta-BDE concentrations in colostrum samples were positively associated with the birth weight of the infants.
6	Zhang et al. [51]	2019	China	Trichloroacetic acid (TCAA)^b^	Systolic pressure, Diastolic pressure, Pulse pressure	Urinary TCAA levels were positively associated with systolic blood pressure (SBP) and pulse pressure based on trend tests after adjusting for potential confounders. Finally, only the association of TCAA with SBP remained significant in the sensitivity analysis. The results suggested that TCAA exposure was associated with increased BP in adults.
7	Zhang et al. [52]	2021	China	Trichloroacetic acid (TCAA) and Dichloroacetic acid (DCAA)^b^	Systolic pressure, Diastolic pressure, Pulse pressure, total cholesterol, platelet indices	Urinary DCAA and TCAA were positively associated with systolic BP. Systolic BP was positively associated with increased Platelet count. DCAA and TCAA might indirectly positively affect platelet count by increasing systolic BP. Urinary DCAA was inversely associated with platelet distribution width even when BP is controlled.
8	Tyagi et al. [44]	2021	India	Hexachlorocyclohexane (HCH) and dichlorodiphenyltrichloroethane (DDT)^c^^**,**^^d^	Case of Type 2 Diabetes (T2DM)	Among all recruited subjects consuming contaminated groundwater, 42 % had T2DM, 38 % pre-diabetes, and the remaining 20 % were found normal. Elevated OCPs level in consumed groundwater may contribute to increased risk for the development of T2DM after a certain period of exposure.
9	Fu et al. [43]	2022	China	Nonylphenol (NP), Bisphenol A (BPA), Dimethyl phthalate (DMP)^c^	Hepatic function indices (direct bilirubin, indirect bilirubin, total bilirubin, Alanine aminotransferase (ALT), Aspartate aminotransferase (AST)).	The association between the exposure and adverse effects on hepatic function was apparent in children. Drinking water was assessed among environmental risk factors, but the use of cosmetics during pregnancy and hair gels & perfumes use by children had a greater impact on hepatic function damage.
10	Deng et al. [53]	2022	China	Trichloroacetic acid (TCAA) and Dichloroacetic acid (DCAA)^b^	Ovarian reserve indicators: Antral follicle count (AFC), ovarian volume, anti-Mullerian hormone (AMH), and follicle-stimulating hormone (FSH).	Urinary TCAA was negatively associated with AFC and AMH. Urinary DCAA was negatively associated with right AFC.
11	Deng et al. [54]	2022	China	Trichloroacetic acid (TCAA) and Dichloroacetic acid (DCAA)^b^	Menstrual cycle: bleeding duration and variation in cycle length	Menstrual function may be affected by exposure to drinking water DBPs. Evidence was observed for the associations of urinary DCAA with prolonged variation in cycle length, as well as urinary TCAA with prolonged bleeding duration.
12	Deng et al. [55]	2023	China	Trichloroacetic acid (TCAA) and Dichloroacetic acid (DCAA)^b^	In Vitro Fertilization Outcomes: Total oocytes and clinical pregnancy	Elevated quartiles of urinary DCAA and TCAA concentrations were associated with reduced numbers of total oocytes and metaphase II oocytes. Urinary DCAA concentration is associated with a lower proportion of best-quality embryos. Older women may be more susceptible to the adverse reproductive effects of DBP exposures.
13	Luo et al. [56]	2023	China	N-Nitrosodimethylamine (NDMA), N-Nitrosodiethylamine (NDEA), N-Nitrosopiperidine (NPIP)^b^	Birth outcome: low birth weight (LBW), small for gestational age (SGA), premature delivery (PTD).	No significant association found between nitrosamines concentration in water on each trimester or averaged entire pregnancy period & the increased risk of LBW. Evidence was found for associations between elevated nitrosamine exposures and reduction in body weight and some increased risks of SGA and PTD.
14	Liu et al. [57]	2024	China	Trichloroacetic acid (TCAA) and Dichloroacetic acid (DCAA)^b^	Case of diminished ovarian reserve (DOR)	In this case-control study among Chinese women undergoing assisted reproductive technology (ART), elevated urinary DCAA levels were associated with higher DOR risk (OR = 1.87). Exposure to drinking water DBPs may contribute to the risk of DOR among women undergoing ART.
15	Li et al. [42]	2024	China	Trichloroacetic acid (TCAA) and Dichloroacetic acid (DCAA)^b^	Kidney function (%changes of Uric Acid (UA) and %changes of Estimated glomerular filtration rate (eGFR)).	A dose-dependent association between elevated urinary DCAA levels and decreased eGFR was observed. There was a difference in age susceptibility for the association between urinary DCAA and UA levels. Urinary DCAA but not TCAA was associated with impaired renal function among women undergoing assisted reproductive technology.

a Specific CECs & Outcome indicators.

b Classified as Disinfection Byproducts (DBPs) in this review.

c Classified as Endocrine Disrupting Chemicals (EDCs) in this review.

d Classified as Pesticide in this review.

Similarly, the concentration of p,p'-DDE, a persistent organic pollutant, showed marked regional differences. In the Asia study, the concentration was 2.49 μg/L, whereas higher levels were observed in both Mexico (6.17 μg/L) [106] and America (20.8 μg/L) [107]. The elevated concentrations in North America suggest a greater historical or ongoing exposure to p,p'-DDE, potentially reflecting variations in agricultural practices or the regulation of pesticides. These findings underscore the need for targeted interventions based on regional exposure patterns to reduce the public health impacts of these environmental contaminants.

The authors opine that the last decade epidemiologic concern of drinking water contaminants of emerging concern (CECs) in Asian Countries are well represented in Table 3. However, in reflecting upon the health outcomes investigated in the studies included in this review (See Fig. 2), it becomes evident that when juxtaposed with the extensive range of potential health outcomes and disease burdens that could emanate from emerging contaminants in drinking water, the health outcomes assessed in the extant studies over the past decade in Asian countries appear to be insufficient. Consequently, the body of literature in this field remains somewhat circumscribed, underscoring the prospective nature of this field, particularly in Asian countries.

The objective of environmental epidemiological studies extends beyond the mere establishment of causality between exposure to environmental contaminants and the manifestation of disease or adverse health outcomes [46]. These studies also serve to elucidate the local context and circumstances, and their role in shaping the distribution, pattern, and determinants of health outcomes, ultimately achieving their aim through the contribution to public health policy [108]. In this regard, the significance and relevance of local or regional epidemiological studies cannot be overstated. Even though investigations into specific emerging contaminants and their health effects may have been conducted elsewhere, the findings of such studies may not fully capture the unique interplay of factors at the local or regional level. Therefore, conducting epidemiological studies in the specific locales where the health impacts are experienced remains of paramount importance. If researchers in these countries, particularly those in the realms of environmental science and public health, are genuinely committed to making a substantive contribution to addressing this issue - with the aim of heightening public awareness and eliciting the attention and action of policy makers - then this field must be prioritized for development. This is not merely about transforming emerging contaminants into a topic for academic review, but rather about fostering a deeper understanding about the present burden of disease and initiating proactive measures to mitigate the potential risks associated with these contaminants in drinking water. In essence, the current state of research in this field underscores the need for a more comprehensive and robust exploration of the potential health impacts of emerging contaminants in drinking water, particularly within the context of Asian countries.

### 4.3 The lack of result & challenges of drinking water CECs in Asia

In this study, the methodology employed for the literature search, encompassing identification, screening, and full-text review conducted by the authors, yielded only 15 peer-reviewed epidemiological studies concerning emerging contaminants in drinking water across Asian countries over the last decade. In the keywords utilized in the search strategy, neither country nor continent names were employed for exclusion or inclusion purposes. Consequently, the screening process to encompass Asian countries was conducted manually by the authors. This approach aimed to minimize the risk of overlooking or excluding potentially relevant studies that might not explicitly mention Asia. Thus, we are confident in reporting the results and concluding that epidemiological research on drinking water CECs has been conducted in only four countries (less than 9 %) in Asia over the last decade. Among the 15 included studies, the majority originate from China (80 %). Only one study was identified for Sri Lanka, the Philippines, and India, respectively. This indicates a significant gap in research and literature addressing this issue suggests that the issue is still being overlooked. The fact that 80 % of the studies originate from China further highlights the uneven distribution of research efforts. While it is commendable that China is leading the way in this research, the lack of studies from other Asian countries is alarming. This is particularly concerning given the unique environmental and socio-economic contexts of each country, which could influence the prevalence and impact of emerging contaminants in drinking water. This gap in research not only hinders our ability to fully comprehend the extent of the problem but also impedes the development of effective mitigation strategies and policies to address it. This stark disparity underscores the need for a more comprehensive and inclusive approach to research in this field.

The presence of emerging contaminants in drinking water poses a multifaceted problem particularly in Asian countries, including developing nations [109,110]. In Southeast Asia especially, the increasing presence of unconventional pollutants in water bodies, soil, and various organisms has become an alarming concern [110]. The distribution of emerging contaminants across Southeast Asian countries is influenced by several factors, including but not limited to population growth, urbanization trends, and agricultural practices [110]. Some of those challenges might apply to other countries in other parts of Asia to some degree as well. The extent and nature of water contamination can vary significantly across different regions in a country, influenced by local industrial activities, agricultural practices, and the adequacy of water treatment facilities [111,112]. In some areas, rapid urbanization and inadequate infrastructure exacerbate the issue, leading to the reliance on contaminated water sources [113,114].

Furthermore, the challenge of addressing emerging contaminants in drinking water is compounded by the lack of, or limited access to, universal healthcare systems in Asian countries. Children, pregnant women, the elderly, and people with compromised immune systems are particularly vulnerable to the adverse effects of contaminated water. In developing countries within Asia, the risk is compounded by limited access to health care. Unsustainable revenue-raising methods, fragmented health insurance schemes, incongruity between insurance benefits and people's needs, political and legislative indifference, and intractable and rapidly rising healthcare cost [115,116] are all some health financing challenges presently faced by Asian countries. In low-income Asian countries especially, the problem might extend to various dimensions of barriers to health care, including geographical access, health care availability, affordability and acceptability [117].

In the Southeast Asia region, a distinct challenge emerges compared to other parts of Asia. Here, rapid industrialization and urbanization have surged ahead, yet they haven't been met with equal measures of awareness and regulatory oversight concerning emerging contaminants [110]. While some countries have developed regulatory frameworks and strategies for some types of pesticides [118] and pathogens like Cryptosporidium [119], there remains a significant lack of awareness and regulatory measures for other emerging contaminants, particularly DBPs and EDCs in this region. This imbalance poses significant risks, particularly in terms of water pollution and associated health hazards. With insufficient regulatory frameworks and limited public awareness campaigns, the situation demands urgent attention.Additionally, climatic conditions such as high temperatures and humidity in this region, combined with lifestyle factors such as occupational and outdoor activities, often lead to increased water consumption [120,121], which can heighten exposure to contaminants present in drinking water. Moreover, varying levels of water literacy across the population affect the understanding and management of water quality risks [122]. It's clear that concerted efforts are needed to address emerging contaminants and protect public health in this region.

Despite these challenges, research on advanced water treatment technologies addressing emerging contaminants has continued to rise. The search and innovation for suitable, cost-effective adsorbent materials is a promising, on-going field of research to treat emerging contaminants in polluted water [123], capable of removing pharmaceuticals and personal-care products [124,125], endocrine disruptors [126], agricultural products, benzene, toluene, and xylene (BTX) products, organic dyes [127], pesticides, and other industrial products [128]. Another treatment alternative, the biological-based technologies such as constructed wetlands, algal-based technologies, enzymatic degradation, and bioreactors are being developed to be capable of removing pharmaceuticals, endocrine disruptors, phenol-based compounds, dyes, inorganic substances, and pesticides [123]. Another well-known water treatment technology is advanced oxidation process (AOP), involving the development and utilization of electrochemical, photochemical and ultrasonic technologies, capable of removing pharmaceuticals, pesticides, dyes, and persistent organic pollutants (POPs) [123,129].

However, without proportional support from a corresponding effort to address CECs from both public and environmental health perspectives, new and advanced treatment technologies may fall short in solving the problem. This is due to the lack of alignment between the wide array of technologies available and the lack of information on actual risk or burden of disease experienced by the community, particularly among drinking water consumers. The successful integration of new technologies hinges on stakeholders’ awareness of emerging contaminants, which are often overlooked and absent from current regulations [130]. In this context, environmental epidemiology studies, a subset of public health and environmental health research, are instrumental in identifying the significant and actual emerging contaminants that pose a health risk to the public. This understanding can be augmented by Health Risk Assessment research, which aids in prioritizing these emerging contaminants. When technological progress is paired with sustained research in this area, specifically in the epidemiology of emerging contaminants, it becomes evident which technologies should be given precedence for adaptation and prioritization within the local context, thereby ensuring their relevance and utility. Furthermore, research in health risk assessment and epidemiology can heighten awareness among regulators and policymakers. This leads to more effective informed environmental and health policy-making, grounded in tangible epidemiological evidence rather than only a review or toxicology studies, which may often still be unfamiliar to local regulators in Asian countries.

### 4.4 Other paramount emerging contaminants in drinking water in Asian countries

While lacking epidemiologic investigation in Asia over the last decade, some types of emerging contaminants are worth discussing here for their emergence, presence, and probably regulation & monitoring negligence in drinking water sources in Asian countries. Emerging contaminants, including (but not limited to) pharmaceuticals & personal care products (PPCPs), microplastics, and per- and poly-fluoroalkyl substances (PFAS), are increasingly found in water bodies due to rapid industrialization and urbanization [109,110]. These contaminants, often present at low concentration levels, can still pose a risk of adverse impacts, especially to vulnerable populations [131]. The main sources of these contaminants include domestic discharges, hospital effluents, industrial wastewaters, runoff from agriculture, livestock and aquaculture, and landfill leachates [109]. Therefore, future research efforts targeting these overlooked emerging contaminants will prove immensely beneficial in informing policymakers and raising public health awareness across Asian countries. One pivotal approach involves conducting rigorous epidemiological studies focused on these contaminants. Some of the latest research about them in Asian countries and their health effects are briefly reviewed here.

#### 4.4.1 Pharmaceuticals and Personal Care Products (PPCPs)

Pharmaceuticals and Personal Care Products (PPCPs) represent a diverse collection of chemical substances used by individuals for personal health or cosmetic reasons and by the agricultural industry to enhance the growth or health of livestock. This category encompasses a wide range of compounds, including, but not limited to, prescription drugs, over-the-counter medications, veterinary drugs, fragrances, and cosmetics [132]. Due to their widespread use and subsequent disposal, PPCPs have been increasingly detected in various environmental compartments, including surface water, groundwater, and even drinking water sources [133], marking them as emerging contaminants of concern. Recent research in Asian countries has confirmed their presence in drinking water sources: in rivers and lakes [133,134], groundwater [135], water treatment plants [136], and even in treated tap water [137].

One of the reasons PPCPs have attracted heightened scientific and regulatory attention is their persistent nature [132,133] and the mechanism of their transport into the aquatic environment. PPCPs enter aquatic environments through various pathways, including the excretion of unmetabolized compounds by humans and animals, indirect disposal of unused medications down sinks and toilets, agricultural runoff, and the discharge of wastewater treatment plants [132,133]. The traditional wastewater treatment plants are not specifically designed to remove PPCPs; hence, their presence is increasingly noted in effluents and, subsequently, in surface and ground waters which are sources of drinking water [135,136,138].

The health impacts of PPCPs in the water are still a subject of ongoing research, primarily due to the complex nature of exposure and the low concentrations typically encountered. However, there is growing evidence to suggest that continuous exposure to certain PPCPs, even at low levels, may lead to adverse health effects, including hormonal disruption, antibiotic resistance, and in the extreme, carcinogenic effects [132]. One particular concern is the endocrine-disrupting chemicals (EDCs) within the PPCP category, which can interfere with the body's endocrine system even at very low concentrations, potentially leading to reproductive and developmental health issues [139].

While the Endocrine Disrupting Chemicals (EDCs) group of emerging contaminants has been discussed in the previous section, it is imperative to highlight the significance of Pharmaceuticals and Personal Care Products (PPCPs). PPCPs encompass a wide range of chemical compounds, each with its unique effects on human and environmental health. Not all PPCPs exhibit endocrine-disrupting properties; instead, they manifest various other effects that warrant thorough investigation. Moreover, the primary route of exposure to PPCPs is through water, mainly originating from wastewater and industrial discharge. Therefore, future epidemiological studies focusing on the diverse array of PPCPs present in water sources will contribute significantly to the understanding of the public health implications associated with these emerging contaminants.

#### 4.4.2 Micro- and nano-plastics

The emergence of micro and nano-plastics as contaminants in drinking water represents a pressing global environmental and public health concern. Defined broadly, microplastics are particles smaller than 5 mm [140], while nanoparticles are even tinier, often measuring from 1 nm to 1 μm [141]. Their ubiquity in water sources poses significant challenges, not only due to their persistent and pervasive nature but also because of their potential to infiltrate the human body with unknown long-term health consequences. Micro and nano-plastics enter water systems through a variety of routes, including runoff from landfills, fragmentation of larger plastic debris, atmospheric deposition, and direct input from industrial and household sources [140]. Once they infiltrate the water cycle, their small size and resistance to biodegradation make them particularly troublesome. Studies have highlighted their ability to adsorb and carry other contaminants, such as heavy metals, organic pollutants, and other emerging contaminants [142], further emphasizing the compound nature of this contamination. Moreover, water treatment facilities, designed primarily for macro-scale particulates, often fail to completely remove these minute particles, allowing them to persist in tap water served to millions [143].

Recent research highlights the significant problems Asian countries face regarding the micro and nano-plastics issue due to rapid urbanization, high population densities, and inadequate waste management systems. In China, microplastics were pervasive in tap water samples across multiple cities, primarily composed of PET and PP particles [144]. Similarly, India's major rivers, such as the Ganges, contain substantial plastic waste, impacting local drinking water sources [145]. Indonesia faces a growing microplastics concern, especially in coastal areas like Jakarta Bay, where PE and PP microplastics are prevalent in both water and sediments [146]. In Malaysia, microplastics are found in various water sources, sediment, and aquatic organisms [147]. The Philippines, with its significant coastline, experiences high microplastic levels in Manila Bay, raising concerns about their ingestion by marine life and subsequent entry into the food chain [148]. South Korea, despite advanced waste management, also grapples with microplastics from agriculture, industry, and domestic sewage in water bodies providing drinking water for millions like the Han River [149].

The health implications of micro and nano-plastics on humans are increasingly becoming a focal point of scientific inquiry. Due to their diminutive size, these particles can easily bypass the body's defensive mechanisms, leading to ingestion and accumulation in various organs. Although the full extent of their impact is still being investigated, there are concerns about their potential to cause oxidative stress, inflammation, and endocrine disruption [150]. Moreover, nano-plastics, owing to their smaller size, pose an even greater threat as they can penetrate cells and tissues, potentially causing DNA damage and other cytotoxic effects [151]. Additionally, as mentioned before, due to their ability to adsorb other contaminants, the health effects of ingesting these tiny, ubiquitous particles should be regarded as compounded. Future epidemiological studies in this area will be significantly valuable to confirm and better understand the multifaceted health effects of micro- and nanoplastics, which are still under extensive investigation.

#### 4.4.3 Per- and polyfluoroalkyl substances (PFAS)

Per- and polyfluoroalkyl substances (PFAS) are synthetic compounds recognized for their persistent environmental pollution. These chemicals are used in products like non-stick cookware, food packaging, and firefighting foams due to their resistance to grease, oil, water, and heat [152]. Their stability, while advantageous for manufacturing, leads to environmental concerns as PFAS do not degrade naturally, earning them the title “forever chemicals” [152]. PFAS contaminate drinking water through industrial discharges, the use of firefighting foams near airports and military bases, and landfill leaching from disposed products containing PFAS [153]. This contributes to their presence in the water cycle, affecting both surface and groundwater sources [154].

The health implications of exposure to PFAS are a growing concern. Research links PFAS to immune system effects, altered cholesterol levels, liver damage, thyroid disruption, and harm to fetal development [155]. Additionally, these compounds are associated with an elevated risk of certain cancers [156]. The detrimental health impacts are believed to be due to PFAS's interaction with the body's protein receptors, disruption of endocrine function, and promotion of oxidative stress [155,156]. As a result, PFAS are recognized as endocrine-disrupting chemicals (EDCs). While the previous section discussed the EDCs group, it is important to note PFAS separately here as a significant yet overlooked group of emerging contaminants in the drinking water of Asian countries.

In Asian countries, the presence of PFAS in drinking water systems and aquatic environments has been increasingly documented, paralleling global trends. For instance, a study conducted in China identified PFAS in the surface water of several major river systems, attributing the contamination primarily to industrial activity and wastewater discharge [157]. In Japan, the situation is similar, with detectable levels of PFAS found in urban rivers and tap water, reflecting both current and historical usage of PFAS-containing products and industrial effluents [158,159]. South Korea has also reported widespread PFAS contamination in drinking water, with research indicating that sources include industrial complexes, military bases, and areas with intensive use of firefighting foams [160]. This list may extend to other Asian countries as well, given the widespread use and disposal of PFAS across the region.

While there is growing documentation and discussion regarding PFAS presence in Asian countries, including detection in drinking water systems and aquatic environments mirroring global trends, the region still lags in comprehensively addressing the health impacts of PFAS contamination in drinking water. During our screening process, a substantial number of epidemiological studies on PFAS in drinking water from American and European countries were identified, reflecting a more advanced understanding and proactive research approach in these regions [161]. The necessity for robust epidemiological studies in Asia is paramount to comprehensively assess and mitigate the potential health risks posed by PFAS exposure, ensuring the development of effective regulatory measures and public health interventions across the continent.

### 4.5 Studies limitations & recommendations

Each of the included studies had unique, notable recommendations regarding future research in this field. There were, however, some limitations and recommendations generally found and stated by the authors which may guide future research development and direction.

#### 4.5.1 Population

A critical recommendation echoed by numerous authors in the reviewed studies pertains to the significance of adequate population size in environmental epidemiology research [44,50]. Larger sample sizes offer greater statistical power, allowing researchers to detect subtle associations between environmental exposures and health outcomes with enhanced precision and reliability. This is particularly crucial in the context of studying contaminants in drinking water, where exposure levels can vary widely across different geographical regions and demographic groups. Robust sample sizes not only strengthen the validity of study findings but also facilitate subgroup analyses, enabling researchers to explore potential susceptibility factors and health disparities within the population.

Moreover, the emphasis on including diverse segments of the general population in epidemiological studies intend to achieve broader applicability and relevance of research findings. Some of the included studies’ populations are hospital/clinic visitors/patients [41,42,47,53,55]. Only a few studies [44,49] recruit the general population with random sampling. Studying the general population allows researchers to capture a more representative sample of individuals across different demographics, lifestyles, and environmental settings. This inclusivity is crucial for identifying varying levels of exposure to contaminants present in drinking water sources and understanding how these exposures may translate into population-wide health outcomes [41,42,50,[53], [54], [55]].

#### 4.5.2 Measurement of CECs in drinking water sources

Of the 15 included studies, only 4 studies (26.66 %) [44,47,49,56] did the exposure assessment by measuring CECs concentration in the water of the drinking water distribution system used by the study population. While many studies in the reviewed literature collected data through participant surveys regarding their drinking water sources, a significant gap exists in quantifying the concentration of Contaminants of Emerging Concern (CECs) directly in the water supply. The measurement of water CECs concentration is of paramount importance for several reasons. Firstly, it provides a more accurate and objective measure of exposure compared to self-reported survey data, thereby reducing potential bias and enhancing the validity of the study findings. Secondly, measuring CEC concentrations is essential for understanding the relationship between exposure and health outcomes. While some contaminants may have a clear dose-response relationship, EDCs may exhibit non-monotonic dose-response patterns. Understanding these patterns is vital to assessing the risk associated with CECs exposure more comprehensively [162,163]. Additionally, measuring these concentrations helps identify whether drinking water is the primary exposure route for these contaminants or if other exposure pathways, not captured in the study, require further investigation.

The inclusion of water sample analysis for CECs concentration not only strengthens the validity of exposure assessments but also provides critical data for risk characterization and regulatory considerations. By quantifying CECs levels in the drinking water systems of study populations, researchers gain valuable insights into the actual environmental exposures faced by individuals. This data-driven approach enables a more nuanced analysis of exposure-outcome relationships, allowing researchers to discern dose-response patterns and evaluate the effectiveness and priority of regulatory measures in mitigating exposure risks. Moving forward, future environmental epidemiology studies should prioritize including direct measurements of CECs concentration in drinking water, to enhance the precision and reliability of study findings and inform evidence-based public health policies.

However, measuring CECs concentration in drinking water presents several technical and logistical challenges that must be considered. The detection and quantification of CECs require advanced analytical methods, such as liquid chromatography coupled with mass spectrometry (LC-MS), which can be both expensive and time-consuming. Furthermore, the variability in CECs' chemical properties necessitates the use of multiple analytical techniques to accurately capture a broad spectrum of contaminants [164]. Additionally, logistical challenges such as the need for extensive sampling networks, the handling and transportation of water samples to centralized laboratories, and the maintenance of sample integrity can pose significant obstacles in large-scale studies. Despite these challenges, future research efforts can overcome these barriers through the development of more cost-effective and robust analytical methods, increased funding for research infrastructure, and the implementation of standardized protocols for sample collection and analysis. By addressing these technical and logistical hurdles, the scientific community can better assess CECs exposure and its potential health impacts, ultimately guiding public health interventions and regulatory policies.

#### 4.5.3 Study design

Cross-sectional studies, while valuable in providing a snapshot view of a population at a specific time, have inherent limitations, particularly in inferring causality. Due to their nature of sampling at a single point in time, they cannot establish the temporal sequence of exposure and outcome. This limitation is critical when investigating complex health effects related to emerging contaminants, as causality requires understanding the exposure preceding the outcome. Another significant challenge associated with cross-sectional studies, especially when measuring biomarkers such as those found in urine or blood, is the potential for fluctuations or wide variability over time [52,54]. This limitation underscores the need for complementary longitudinal studies that can track biomarker levels over time and account for temporal variations. Recognizing this limitation, several authors of the included studies [[41], [42], [43],^51^,^52^,^54^,^57^] noted that the results of their studies were to be interpreted carefully and emphasized the importance of robust sampling and assessment methods. They recommend complementing cross-sectional data with longitudinal studies to capture changes over time and establish potential causal relationships.

Future epidemiological research in this area must carefully consider the limitations of cross-sectional designs and work towards incorporating more robust methodologies. Longitudinal studies, which track individuals or populations over time, offer a promising approach to elucidate causal relationships between exposure to emerging contaminants and health outcomes. Additionally, researchers should aim to compare and integrate findings from cross-sectional studies with other study designs, such as case-control or cohort studies, to strengthen the evidence base and provide a more comprehensive understanding of the health effects associated with exposure to contaminants in drinking water.

In addition to these methodological improvements, future research should also focus on employing mixed-methods approaches that integrate quantitative and qualitative data to better understand the contexts and factors affecting exposure to CECs from drinking water intake. Utilizing geospatial analysis to map exposure hotspots and advanced statistical models to control for confounding variables could also enhance the robustness of study findings. Moreover, expanding research to include multi-site studies across diverse geographic regions would provide broader insights into regional disparities and unique challenges, thereby facilitating more targeted public health interventions. Collaborative efforts involving multiple disciplines and stakeholders, including policymakers, could ensure that research findings translate into effective regulatory actions and public health strategies.

## 5 Review conclusion, strength & limitations

### 5.1 Conclusion

A rigorous database search has revealed a paucity of epidemiological studies on emerging contaminants in drinking water in Asian countries. Over the past decade (2014–2024), only 15 studies have been conducted, originating from a mere four countries - China, India, Sri Lanka, and the Philippines. This represents less than 9 % of Asian countries, underscoring both the research gap and the potential for future exploration in this area. The Contaminants of Emerging Concern (CECs) assessed in these studies primarily include Disinfection Byproducts (10 studies), Endocrine Disruptors (2 studies), Pesticides (2 studies), and one Protozoan Pathogen. However, when comparing each of these emerging contaminants, particularly the chemicals with their unique potential health effects, it becomes evident that the health outcomes assessed in the existing studies are rather inadequate. They lack the breadth necessary to capture and address the wide potential burden of disease that the CECs in drinking water may cause.

This review also highlights three more emerging contaminants in Asian countries (PPCPs, micro- and nanoplastics, and PFAS) that future research in this area should address, considering their ubiquitous nature and the current lack of research. To effectively bring the issue of CECs to the forefront, it is essential to move beyond academic discussions. The ultimate aim of epidemiological studies is to contribute to informed policy-making and raise awareness among both the public and policymakers. Therefore, researchers in Asian countries, particularly in the field of environmental and public health, should consider developing this area of research. They should take note of the recommendations made by previous studies, such as those included in this review, and expand both the types of CECs and health outcomes assessed to cover the overlooked contaminants and diseases that are not yet explored. This approach will significantly contribute to our understanding of the impact of CECs on public health and guide the development of effective strategies to mitigate their effects.

### 5.2 Strength & limitations

Although there have been numerous original research and reviews of emerging contaminants, their presence, their health effects, and risk assessment in Asian countries, to our knowledge this is the first attempt to review the epidemiology study regarding the wide categories of CECs in Asian countries. This scoping review represents the first comprehensive attempt to evaluate epidemiological studies concerning a broad spectrum of CECs in Asian countries. By addressing this critical gap in research, our review serves as the initial stride toward developing a robust body of literature focused on addressing CEC-related challenges in Asian drinking water systems. As a scoping review, our work also provides valuable insights into the feasibility of conducting future systematic reviews in this domain. The current findings indicate a scarcity of available literature, suggesting that a systematic review might face challenges in terms of data availability specific to Asian countries. However, given the overarching aim of systematic reviews to address global health burdens comprehensively, overcoming regional constraints and encompassing CECs as a worldwide health concern in drinking water systems might be feasible for future direction.

This review focused on capturing all drinking water contaminants that is considered as emerging contaminants/contaminants of emerging concern in drinking water systems using specific keywords like “emerging” along with typically well-known CECs types such as DBPs, EDCs, PPCPs, and microplastics/nanoplastics. However, studies evaluating specific contaminants within these categories that do not explicitly label them as emerging contaminants or within the CEC types outlined in our search strategy might have been missed. Consequently, relevant studies that did not overtly identify as part of this subset could have been inadvertently excluded, potentially leading to gaps in the review's coverage.

Additionally, The highly targeted nature of our review, emphasizing epidemiological designs, emerging contaminants, the context of drinking water, and focusing on Asian countries, inherently limited the inclusion of studies that closely intersect with these criteria. While this precision ensures a detailed exploration of a specific area of study, it might have overlooked broader discussions or related research topics tangential to our core focus. Therefore, the review's findings highlight the lack of research emphasis within this specified domain rather than indicating a definitive lack of awareness or concern regarding CECs in drinking water across Asian countries. It is crucial to interpret these limitations within the context of our review's defined objectives and scope.

## CRediT authorship contribution statement

**Rinaldy Jose Nathanael:** Writing – review & editing, Writing – original draft, Visualization, Validation, Software, Resources, Project administration, Methodology, Investigation, Formal analysis, Data curation, Conceptualization. **Latonia Nur Adyanis:** Writing – review & editing, Writing – original draft, Visualization, Validation, Software, Resources, Project administration, Methodology, Investigation, Formal analysis, Data curation, Conceptualization. **Katharina Oginawati:** Writing – review & editing, Visualization, Validation, Supervision, Software, Resources, Project administration, Methodology, Investigation, Formal analysis, Data curation, Conceptualization.

## Availability of data and material

The data and materials used in this study are available from the corresponding author upon reasonable request.

## Funding

The authors declare that no funds, grants, or other support were received during the preparation of this manuscript.

## Declaration of competing interest

The authors declare that they have no known competing financial interests or personal relationships that could have appeared to influence the work reported in this paper.
