# Supplementary material for: The last decade epidemiologic concern of drinking water contaminants of emerging concern (CECs) in Asian Countries: A scoping review
Source: Heliyon. 2024 Oct 12;10(20):e39236. doi: 10.1016/j.heliyon.2024.e39236 (PMC11620247; doi:10.1016/j.heliyon.2024.e39236)
Supplement: Multimedia component 1 [file mmc1.pdf]

Table A1. Average CECs concentration reported from included studies.

No	Contaminants of Emerging Concern (CECs)			Drinking Water	Urine	Blood	Colostrum
1	Disinfection Byproducts (DBPs)	Haloacetic Acids (HAA)	Trichloroacetic Acid (TCAA)		9.58 μg/L[1]		
					6.41 μg/g Creat.[2]		
					6.09 μg/g Creat.[3]		
					6 μg/L[a] [4]		
					5.07 μg/L[a] [5]		
					5.98 μg/L[a] [6]		
					5.05 μg/L[a] [7]		
					5.04 μg/L[a] [8]		
			Dichloroacetic Acid (DCAA)		5.34 μg/g Creat. [3]		
					5.03 μg/L[a] [4]		
					4.58 μg/L[a] [5]		
					6.76 μg/L[a] [6]		
					5.41 μg/L[a] [7]		
					4.58 μg/L[a] [8]		
		Trihalomethanes (THM)	Chloroform			40.7 ng/L [9]	
			Bromodichloromethane			1.5 ng/L [9]	
			Dibromochlormethane			0.9 ng/L [9]	
			Bromoform			1.6 ng/L [9]	
			Total THM			52.3 ng/L [9]	
		Nitrosamines	N-nitrosodimethylamine (NDMA)	18.6 ng/L [10]			
			N-nitrosodiethylamine (NDEA)	3.6 ng/L [10]			
			N-nitrosopiperidine (NPIP)	2.9 ng/L [10]			
			Total Nitrosamines	26.1 ng/L [10]			

2	Endocrine Disrupting Chemicals (EDCs)	Polybrominated Diphenyl Ethers (PBDE)	Total tri- to hexa- BDE				9.1 ng/g lipid weight[b] [11]
			Nonylphenol (NP)			45.85 ng/mL[b,c] [12]	
			Bisphenol-A (BPA)			26.31 ng/mL[b,c] [12]	
			Dimethyl phthalate (DMP)			31.62 ng/mL[b,c] [12]	
3	Pesticides	Herbicides	Glyphosate	Abandoned well: 3.5 μg/L [13]			
				Serving well: 0.7 μg/L [13]			
				Surface water: 0.05 μg/L [13]			
		Organochlorine pesticides	α-Hexachlorocyclohexane (α-HCH)	0.005 μg/L[e] [14]		1.36 μg/L[e] [14]	
			β-Hexachlorocyclohexane (β-HCH)	0.043 μg/L[e] [14]		2.36 μg/L[e] [14]	
			γ-Hexachlorocyclohexane (γ-HCH)	0.696 μg/L[e] [14]		1.66 μg/L[e] [14]	
			o,p’-Dichlorodiphenyltrichloroethylene (DDE)[d]	0.031 μg/L[e] [14]		0.9 μg/L[e] [14]	
			p,p’-DDE[d]	0.558 μg/L[e] [14]		2.49 μg/L[e] [14]	
			p,p’-Dichlorodiphenyldichloroethane (DDD)[d]	0.025 μg/L[e] [14]		1.05 μg/L[e] [14]	

			o,p' Dichlorodiphenyltrichloroethane (DDT)[d]	1.01 µg/L[e] [14]		2.28 µg/L[e] [14]	
			p,p'DDT[d]	0.078 µg/L[e] [14]		0.94 µg/L[e] [14]	
4	Protozoan Pathogen	Cryptosporidium	Cryptosporidium Oocysts	River: 0.4 Oocysts/L [15]			
				Creek: 0.8 Oocysts/L [15]			
				Pump: 0.1 Oocysts/L [15]			

Note:

[a]Adjusted by urine specific gravity

[b]Median concentration

[c]Blood serum

[d]Isomers

[e]Water & blood sample from Type 2 Diabetes case group

References

1. Zeng Q, Wang YX, Xie SH, et al. Drinking-Water Disinfection By-products and Semen Quality: A Cross-Sectional Study in China. *Environ Health Perspect*. 2014;122(7):741-746. doi:10.1289/ehp.1307067

2. Zhang S hui, Guo A jing, zhao W xin, Gu J ling, Zhang R, Wei N. Urinary trichloroacetic acid and high blood pressure: A cross-sectional study of general adults in Shijiazhuang, China. *Environ Res*. 2019;177:108640. doi:10.1016/j.envres.2019.108640

3. Zhang S hui, Guo A jing, Wei N, Zhang R, Niu Y jie. Associations of urinary dichloroacetic acid and trichloroacetic acid exposure with platelet indices: Exploring the mediating role of blood pressure in the general population. *J Hazard Mater*. 2021;402:123452. doi:10.1016/j.jhazmat.2020.123452

4. Deng YL, Luo Q, Liu C, et al. Urinary biomarkers of exposure to drinking water disinfection byproducts and ovarian reserve: A cross-sectional study in China. *J Hazard Mater*. 2022;421:126683. doi:10.1016/j.jhazmat.2021.126683

5. Deng YL, Luo Q, Yuan XQ, et al. Associations between drinking water disinfection byproducts and menstrual cycle characteristics: A cross-sectional study among women attending an infertility clinic. *Int J Hyg Environ Health*. 2022;241:113931. doi:10.1016/j.ijheh.2022.113931

6. Deng YL, Liu C, Yuan XQ, et al. Associations between Urinary Concentrations of Disinfection Byproducts and *in Vitro* Fertilization Outcomes: A Prospective Cohort Study in China. *Environ Health Perspect*. 2023;131(9). doi:10.1289/EHP12447

7. Liu XY, Zhang M, Gu XL, et al. Urinary biomarkers of drinking-water disinfection byproducts in relation to diminished ovarian reserve risk: A case-control study from the TREE cohort. *Science of The Total Environment*. 2024;912:168729. doi:10.1016/j.scitotenv.2023.168729

8. Li CR, Deng YL, Miao Y, et al. Exposures to drinking water disinfection byproducts and kidney function in Chinese women. *Environ Res*. 2024;244:117925. doi:10.1016/j.envres.2023.117925

9. Cao WC, Zeng Q, Luo Y, et al. Blood Biomarkers of Late Pregnancy Exposure to Trihalomethanes in Drinking Water and Fetal Growth Measures and Gestational Age in a Chinese Cohort. *Environ Health Perspect*. 2016;124(4):536-541. doi:10.1289/ehp.1409234

10. Luo Q, Miao Y, Liu C, et al. Maternal exposure to nitrosamines in drinking water during pregnancy and birth outcomes in a Chinese cohort. *Chemosphere*. 2023;315:137776. doi:10.1016/j.chemosphere.2023.137776

11. Yin S, Guo F, Aamir M, Liu Y, Tang M, Liu W. Multicenter biomonitoring of polybrominated diphenyl ethers (PBDEs) in colostrum from China: Body burden profile and risk assessment. *Environ Res*. 2019;179:108828. doi:10.1016/j.envres.2019.108828

12. Fu X, He J, Zheng D, et al. Association of endocrine disrupting chemicals levels in serum, environmental risk factors, and hepatic function among 5- to 14-year-old children. *Toxicology*. 2022;465:153011. doi:10.1016/j.tox.2021.153011

13. Jayasumana C, Paranagama P, Agampodi S, Wijewardane C, Gunatilake S, Siribaddana S. Drinking well water and occupational exposure to Herbicides is associated with chronic kidney disease, in Padavi-Sripura, Sri Lanka. *Environmental Health*. 2015;14(1):6. doi:10.1186/1476-069X-14-6

14. Tyagi S, Siddarth M, Mishra BK, Banerjee BD, Urfi AJ, Madhu SV. High levels of organochlorine pesticides in drinking water as a risk factor for type 2 diabetes: A study in north India. *Environmental Pollution*. 2021;271:116287. doi:10.1016/j.envpol.2020.116287

15. Labana R V., Dungca JZ, Nissapatorn V. Community-based surveillance of Cryptosporidium in the indigenous community of Boliwong, Philippines: from April to December 2017. *Epidemiol Health*. 2018;40:e2018047. doi:10.4178/epih.e2018047
